# Which Procedure Yields Better Outcomes: Sleeve Gastrectomy, Roux-en-Y Gastric Bypass or Mini Gastric Bypass? Seven Years Outcome Analysis

**DOI:** 10.3390/medicina61030442

**Published:** 2025-03-01

**Authors:** Ahmet Can Sari, Mehmet Alperen Avci, Sonmez Ocak, Can Akgun, Omer Faruk Buk, Ahmet Burak Ciftci, Emin Daldal

**Affiliations:** 1 Samsun Gazi Hospital General Surgery, 55070 Samsun, Turkey; dr.ahmetcansari@gmail.com; 2 Department of General Surgery, Faculty of Medicine, Samsun University General Surgery, 55090 Samsun, Turkey; canakgun@outlook.com.tr (C.A.); drburakciftci@yahoo.com (A.B.C.); emindaldal@hotmail.com (E.D.); 3 Samsun Medicana Hospital General Surgery, 55080 Samsun, Turkey; sonmezdr@gmail.com (S.O.); omerfarukbuk@gmail.com (O.F.B.)

**Keywords:** bariatric surgery, comorbidities, complications, remission, long term

## Abstract

*Background and Objectives:* Bariatric surgery is the most effective method for achieving sustainable weight loss, improving quality of life, and resolving obesity-related comorbidities over the long term. However, data from long-term studies remain scarce and contradictory. *Materials and Methods:* This study is a retrospective analysis of prospectively collected data over a 7-year follow-up period involving 211 patients diagnosed with morbid obesity who underwent sleeve gastrectomy (SG), Roux-en-Y gastric bypass (RYGB), or mini gastric bypass (MGB) at Samsun University Training and Research Hospital, Department of General Surgery, between 1 January 2014 and 1 January 2018. Changes in weight, remission of associated comorbidities, postoperative complications, re-admission rates, and revision requirements were compared among the patients. *Results:* Of the 211 patients, 20.4% were male, and 79.6% were female. During the study period, 61.1% of patients underwent SG, 29.4% underwent MGB, and 9.5% underwent RYGB. There was no statistically significant difference among the three surgical techniques in terms of weight change parameters, comorbidity remission, postoperative complications, and readmission rates. However, revision rates were significantly higher among patients who underwent SG (*p* < 0.05). *Conclusions:* SG, MGB, and RYGB techniques are comparable and reliable methods in terms of long-term weight loss, surgical outcomes, and complications. After a 7-year follow-up period, all three techniques were found to be similar in terms of HT, T2DM, and GERD remission; however, SG was observed to have a higher revision requirement compared to the other surgical techniques.

## 1. Introduction

Obesity is a prevalent and preventable public health issue worldwide. According to the World Health Organization (WHO) in 2022, approximately 890 million adults aged 18 and above are living with obesity worldwide. These figures, which represent more than a twofold increase compared to 1990, underscore the well-established link between obesity and adverse health outcomes. Furthermore, according to WHO, nearly 2.8 million deaths globally each year are attributed to being overweight or obese [1]. The management of obesity includes balanced diet recommendations, lifestyle modifications, and adjunctive therapies with pharmacological agents, such as Saxenda and Mysimba [2]. Nevertheless, bariatric surgery may be recommended for resistant patients who meet specific criteria established by regulatory bodies such as the National Health Service (NHS) (e.g., BMI ≥ 40 kg/m^2^ or BMI ≥ 35 kg/m^2^ with potential obesity-related comorbidities, failure of conservative management, willingness to adhere to long-term follow-up, and no contraindications for general anesthesia) [3]. Bariatric surgery is the most effective method for achieving sustainable weight loss, improving quality of life, and resolving obesity-related comorbidities. With advances in technology, it is now performed using a variety of techniques [4].

The choice of surgical technique is influenced by patient-specific factors such as dietary habits and comorbidities, as well as the surgeon’s preference, with each technique having its advantages and disadvantages [5]. Sleeve gastrectomy (SG) has become the most commonly performed bariatric surgical technique in recent years, gaining prominence due to its short learning curve, low leakage rates, effective excess weight loss (EWL), resolution of comorbidities, and the advantages it offers for transitioning to other surgical techniques. However, it is also associated with a higher incidence of obesity relapse, gastroesophageal reflux disease (GERD)-related esophagitis, and Barrett’s esophagus [6,7]. Roux-en-Y gastric bypass (RYGB) is a widely accepted and effective method for achieving significant weight loss and improving comorbidities. Studies comparing postoperative outcomes of RYGB and SG have demonstrated similar results in weight loss, Type 2 diabetes mellitus (T2DM), dyslipidemia, and quality of life, although RYGB has been shown to be more effective in hypertension remission and weight loss [8,9]. Mini gastric bypass (MGB), also known as single-anastomosis or omega gastric bypass, has become increasingly popular in recent years. It is characterized by shorter operative times, lower morbidity and mortality rates, and superior or equivalent outcomes to RYGB in T2DM remission and EWL. However, it is also associated with adverse outcomes such as bile reflux, marginal ulcers, and remnant gastric cancer [10,11].

A review of the literature reveals that most studies compare two surgical techniques, with limited research available on the comparison of SG, RYGB, and MGB. Therefore, in this study, we aimed to compare the efficacy and medium-term postoperative outcomes of SG, RYGB, and MGB in patients undergoing surgery for obesity and associated comorbidities.

## 2. Materials and Methods

This study is a retrospective analysis of prospectively collected data from 211 out of 289 patients diagnosed with morbid obesity who underwent sleeve gastrectomy (SG), Roux-en-Y gastric bypass (RYGB), or mini gastric bypass (MGB) at the Department of General Surgery, Samsun University Training and Research Hospital, between 1 January 2014, and 1 January 2018. The study is derived from a thesis, approved by the local ethics committee (approval number: GOKAEK 4/2/2024), and conducted in accordance with the principles of the Declaration of Helsinki. Patients included in the study were over 18 years of age, had a BMI ≥ 40 kg/m^2^ or BMI ≥ 35 kg/m^2^ with associated obesity-related comorbidities, and had completed a 7-year follow-up period post-surgery. Seventy-eight patients who did not attend regular follow-ups, refused the procedure, or did not provide written informed consent were excluded from the study.

All patients underwent a 6-month preoperative evaluation, and the indication for bariatric surgery was determined by a multidisciplinary team. A comprehensive preoperative assessment included patient history, physical examination, laboratory testing, and consultations with relevant specialties. Comorbidities such as diabetes, hypertension, hyperlipidemia, asthma, sleep apnea, and gastroesophageal reflux disease (GERD) were documented in a database. Written informed consent was obtained from all patients. Patients were routinely scheduled for follow-up visits on postoperative day 15 and at 1, 3, 6, and 12 months during the first year. In subsequent years, follow-ups were conducted biannually, during which metabolic parameters and weight changes were recorded. Data collected included age, gender, height, weight, comorbidities (type 2 diabetes mellitus (T2DM), hypertension (HT), asthma, sleep apnea), and laboratory parameters (HbA1c, HDL, LDL, cholesterol, triglycerides, and insulin) through the hospital information system. Patients were followed for 7 years. At the end of 7 years, weight change rates and percentages (EWL [excess weight loss] (%) final, EWL (%) lowest, TWL [total weight loss] (%) final, TWL (%) lowest, BMI change–final, BMI change–lowest, BMI loss (%) final, BMI loss (%) lowest, weight change), changes in lipid profiles, remission rates of T2DM, HT, and GERD, postoperative complications, hospital readmissions, and revision requirements were compared. The term “final” refers to the weight change achieved at the end of the follow-up period, whereas “lowest” refers to the lowest body weight reached during the follow-up period.

In this study, remission of obesity-related metabolic disorders was defined as follows: systolic blood pressure below 140 mm Hg and discontinuation/reduction of antihypertensive medication for HT remission; HbA1c < 6.5 without medication use for T2DM remission; and resolution of postprandial chest burning, regurgitation, and reduced reflux symptoms confirmed by endoscopic findings for GERD remission.

All bariatric procedures were performed laparoscopically and standardized. The SG procedure involved dissection starting 4–6 cm proximal to the pylorus and extending along the greater curvature up to the angle of His. Fat tissue at the esophagogastric junction was removed, and the left hiatal crus was fully exposed. A 36 French orogastric tube was inserted, and sleeve gastrectomy was completed using an endoscopic stapler guided by the tube. The RYGB procedure consisted of a 30 cc gastric pouch, a 150 cm alimentary limb, and a 50 cm biliary limb. Mesenteric defects were closed. The MGB procedure involved a long gastric tube calibrated with a 36 French bougie starting at the incisura angularis. A single gastrojejunal anastomosis was performed using a linear stapler, with a 200 cm biliopancreatic limb.

## 3. Statistical Analysis

Data analysis was performed using SPSS v28 and R Studio software 4.4.0 packages. The normality of the data distribution was assessed using the Kolmogorov–Smirnov Test and Skewness–Kurtosis values. For continuous variables following a normal distribution, mean and standard deviation values were calculated. The Mann–Whitney U test was used to analyze differences between two independent nonparametric groups, while the Student’s t-test was applied for two independent parametric groups. The Wilcoxon test was used to evaluate differences between two dependent nonparametric measurements. For comparisons among more than two groups, the Kruskal–Wallis test was used for nonparametric data, and the ANOVA test was used for parametric data. Post hoc analyses were assessed using Bonferroni-corrected *p*-values. Frequency distributions were provided for categorical variables, and the Chi–square test was applied. Correlations between two nonparametric continuous variables were evaluated using Spearman’s correlation test. A *p*-value of less than 0.05 was considered statistically significant.

## 4. Results

A total of 211 patients meeting the study criteria were included, of whom 43 (20.4%) were male, and 168 (79.6%) were female. Among the patients, 129 (61.1%) underwent SG, 62 (29.4%) underwent MGB, and 20 (9.5%) underwent RYGB (Table 1).

No significant association was observed between type of operation and weight change (Table 2).

When analyzing comorbidity remission and revision based on patients type of operation, no significant difference was observed in terms of HT, T2DM, and GERD remission. SG was significantly associated with a higher need for revision surgery (*p* = 0.0372) (Table 3).

When comparing preoperative and postoperative lipid and diabetes parameters, no significant difference was found in triglyceride levels for the RYGB procedure and insulin levels for the MGB procedure, whereas all other values showed significant results (Table 4). Additionally, no significant differences were observed in preoperative and postoperative lipid and diabetes parameters among the different types of operations (Table 5).

No significant differences were observed in postoperative complications or readmissions based on type of operation (Table 6).

## 5. Discussion

Metabolic and bariatric surgery is currently the most effective method for treating morbid obesity and obesity-related comorbidities, such as type 2 diabetes mellitus (T2DM). It has been proven effective not only in achieving short-term weight loss but also in maintaining reduced body weight in the long term. The success of bariatric surgery is defined as achieving a loss of 50–70% of excess weight (EWL), a reduction of 20–30% of initial body weight, or attaining a BMI < 35 kg/m^2^ [12]. Although some authors have recently advocated abandoning %EWL and %BMI loss values, weight loss outcomes are traditionally still reported using %EWL, %BMI loss, and BMI change. Since 2015, the International Obesity Association has strongly recommended reporting absolute changes in BMI and %TWL for weight loss outcomes [13]. In the literature, the SLEEVEPASS study, which includes 7-year follow-up results, reported no significant differences in %EWL and quality of life between RYGB and SG [8]. A meta-analysis comparing MGB and SG found that MGB had significantly higher %EWL [14], while another meta-analysis reported MGB to be more effective than SG in weight loss and comorbidity remission [15]. Jammu et al. [16] compared 1107 patients who underwent SG, RYGB, and MGB and found that %EWL was statistically significantly higher in MGB after 7 years. In our study, no significant differences were found among RYGB, MGB, and SG in terms of weight change, %EWL, %TWL, or BMI loss.

Weight loss has long been known to be an effective treatment for hypertension. However, the role of bariatric surgical procedures in the treatment of isolated hypertension is less clear. Although early postoperative improvements in hypertension have been observed, long-term outcomes remain variable. Peterli et al. [17] reported HT remission rates of 62.2% and 75.5% for RYGB and SG, respectively, in patients with a 5-year follow-up, with no statistically significant differences. Similarly, Tovar et al. [11] reported comparable HT remission rates for SG and RYGB at 5 years. The SLEEVEPASS study also demonstrated a significant superiority of RYGB over SG in HT remission [8]. Ebadinejad et al. [18] found no significant differences in remission rates among bariatric procedures in a prospective cohort of 787 patients undergoing MGB and LSG. In our study, no significant differences were found among the three surgical procedures regarding HT remission.

Bariatric surgery is widely recognized as a highly effective treatment for T2DM in obese patients. Comparing the effectiveness of commonly performed bariatric procedures on T2DM remission is of vital importance. A cohort study of 9710 patients undergoing SG or RYGB reported better glycemic control, higher diabetes remission rates, and lower recurrence rates for RYGB in both the short and long term [19]. However, some meta-analyses have shown comparable antidiabetic outcomes for SG and RYGB after five years [20,21]. Tovar et al. [11] reported MGB to be superior to SG and RYGB in T2DM remission, with SG and RYGB showing similar remission rates. In contrast, some studies have found no statistically significant differences in T2DM remission among SG, MGB, and RYGB [22,23]. In our study, no statistically significant difference was found among the three surgical techniques in terms of T2DM remission. Obesity is a significant risk factor for gastroesophageal reflux disease (GERD). While weight loss and lifestyle changes can reduce GERD symptoms, the effects of different bariatric procedures on these symptoms vary. Many studies have reported increased intragastric pressure, reflux episodes, esophageal stasis, and acid and non-acid reflux following SG, with higher reflux rates compared to RYGB [24,25]. In our study, no significant differences were observed among the surgical types; however, GERD incidence was significantly higher in patients over 40 years of age (*p* = 0.047). Revision surgery following obesity relapse or severe malnutrition after bariatric procedures is a critical issue. Lazzati et al. [26] reported SG revision rates of 4.7%, 7.5%, and 12.2% at 5, 7, and 10 years, respectively, in a 10-year follow-up study. Studies have also reported revision rates of approximately 1–4% after MGB due to obesity relapse, marginal ulcers, or severe malnutrition [4,27,28]. In our study, SG was significantly associated with a higher need for revision surgery (*p* = 0.0372).

Significant improvements in serum lipid concentrations are observed following metabolic and bariatric surgery. Many studies have reported comparable HDL levels for RYGB and SG in the short and medium term [24,29]. Gu et al., in a meta-analysis involving SG and RYGB, found no significant differences in treatment effects on HDL and triglycerides after five years [30]. The SM-BOSS study reported significant improvements in total cholesterol, HDL, total cholesterol/HDL ratio, LDL, and triglycerides in both SG and RYGB groups after 5 years, with no significant differences between the groups in total cholesterol, HDL, and triglycerides [17]. In our study, significant improvements were observed in HDL, LDL, cholesterol, and triglycerides after 7 years; however, no significant differences were found among the surgical types regarding lipid parameters.

This study has several limitations. It was designed as a retrospective and single-center study. The retrospective nature limits the ability to establish causality and introduces potential bias. While a prospective study allows for more controlled and systematic data collection, our retrospective approach relied on existing records. Another major limitation was the reliance on clinical evaluation for reflux symptoms, with pH monitoring and esophageal manometry studies performed in only a small number of patients.

## 6. Conclusions

Our study demonstrated that SG, MGB, and RYGB are effective, safe, and beneficial techniques in metabolic and bariatric surgery. After a 7-year follow-up period, all three techniques were found to be comparable in terms of HT, T2DM, and GERD remission. However, SG exhibited a higher revision requirement compared to the other surgical techniques. Patient characteristics and the surgeon’s preference are the most critical parameters in the choice of surgical technique. Further multicenter clinical studies with longer follow-up durations and larger patient populations are required to provide more robust evidence in this area.

## Figures and Tables

**Table 1:** Characteristics of patients based on gender, age, type of surgery, and comorbid disease.

		n	%
Gender	Male	43	20.4
	Female	168	79.6
Age	<20	2	0.9
	20–39	106	50.2
	40–59	98	46.4
	>60	5	2.4
Type of surgery	RNYGB	20	9.5
	MGB	62	29.4
	SG	129	61.1
Comorbid disease	Asthma	16	7
	Sleep apnoea	32	15
	Hypertension	44	20
	Type 2 diabetes mellitus	62	29

RNYGB: Roux-en-Y gastric bypass; MGB: mini gastric bypass; SG: sleeve gastrectomy.

**Table 2:** Comparison of weight change parameters based on type of surgery.

	Type of Surgery			*p*
	RNYGB	MGB	SG	
	(n = 20)	(n = 62)	(n = 129)	
EWL (%) (Final)	77.917 ± 21.325	77.663 ± 30.551	71.114 ± 27.807	0.253
EWL (%) (Lowest)	94.854 ± 17.161	95.458 ± 24.162	92.081 ± 19.509	0.544
TWL (%) (Final)	34.862 ± 10.442	34.770 ± 13.240	32.543 ± 12.494	0.448
TWL (%) (Lowest)	42.186 ± 8.331	42.670 ± 9.571	42.065 ± 8.701	0.908
BMI change (Final)	13.001 ± 4.011	12.669 ± 5.046	11.933 ± 4.539	0.445
BMI change (Lowest)	16.123 ± 5.883	16.155 ± 6.475	15.433 ± 6.401	0.731
BMI loss (%) (Final)	34.862 ± 10.442	34.770 ± 13.240	32.543 ± 12.494	0.448
BMI loss (%) (Lowest)	42.186 ± 8.331	42.670 ± 9.571	42.065 ± 8.701	0.908
Weight change	43.35 ± 15.57	44.57 ± 17.54	42.49 ± 18.83	0.759

EWL: excess weight loss; TWL: total weight loss; BMI: body mass index. The data are presented as mean ± standard deviation. Statistical tests: Independent samples *t*-test and one-way analysis of variance (ANOVA).

**Table 3:** Comparison of comorbidity remission and revision requirements based on type of surgery.

		HT Remission		*p*	T2DM Remission		*p*	GERD Remission		*p*	Revision Requirement		*p*
		Present	Absent		Present	Absent		Present	Absent		Present	Absent	
Type of Surgery	RNYGB	4 (9)	1 (8.3)	0.202	6 (9.6)	1 (25.0)	0.564	1 (2)	19 (11.7)	0.126	0	20 (10.3)	0.0372
	MGB	17 (38.6)	4 (33.3)		27 (43.5)	1 (25.0)		16 (32.7)	46 (28.4)		2 (11.1)	60 (31.1)	
	SG	23 (52.2)	7 (58.3)		29 (46.7)	2 (50.0)		32 (65.3)	97 (59.9)		16 (88.9)	113 (58.6)	

HT: hypertension; T2DM: type 2 diabetes mellitus: GERD: gastroesophageal reflux disease. The data are presented as n (%). Test: Chi-square test.

**Table 4:** Comparison of lipid profile and diabetes parameters based on type of surgery.

			HbA1c	HDL	LDL	Cholesterol	Triglycerides	Insulin
Type of Surgery	**RNYGB**	**Preop**	6.32 ± 1.86	45.25 ± 9.15	129.86 ± 31.06	204.8 ± 33.81	166.05 ± 54.84	38.13 ± 41.62
		**Postop**	5.7 ± 0.89	57.08 ± 12.61	116.68 ± 31.17	196.13 ± 41.21	110.19 ± 50.7	9.5 ± 5.45
		***p***	<0.001 *	<0.001 *	0.021 *	0.005 *	0.090	<0.001 *
	**MGB**	**Preop**	6.08 ± 1.17	47.02 ± 11.72	129.53 ± 27.91	210.07 ± 31.19	188.26 ± 101.59	37.93 ± 39.66
		**Postop**	5.39 ± 0.84	57.5 ± 13.84	105.72 ± 29.95	179.64 ± 38.93	101.85 ± 52.47	10.57 ± 6.39
		***p***	<0.001 *	<0.001 *	0.002 *	<0.001 *	<0.001 *	0.134
	**SG**	**Preop**	6.46 ± 1.7	46.36 ± 13.44	127.73 ± 39.17	210.47 ± 50.08	198.66 ± 106.71	44.37 ± 47.31
		**Postop**	5.51 ± 0.81	57.06 ± 15.12	117.94 ± 45.54	194.06 ± 54.34	108.92 ± 49.4	11.88 ± 8.24
		***p***	<0.001 *	<0.001 *	<0.001 *	<0.001 *	<0.001 *	<0.001 *

**HDL:** high-density lipoprotein; **LDL:** low-density lipoprotein. The data are presented as mean ± standard deviation. ***p*:** paired samples *T*-test and one-way analysis of variance (ANOVA) * *p*-value is significant at the 0.05 level.

**Table 5:** Comparison of lipid profile and diabetes parameters based on the type of surgery.

	Type of Surgery						*p* (Pre)	*p* (Post)
	RNYGB (n = 20)		MGB (n = 62)		SG (n = 129)			
	Pre	Post	Pre	Post	Pre	Post		
**HbA1c**	6.32 ± 1.86	5.7 ± 0.89	6.08 ± 1.17	5.39 ± 0.84	6.46 ± 1.7	5.51 ± 0.81	0.308	0.329
**HDL**	45.25 ± 9.15	57.08 ± 12.61	47.02 ± 11.72	57.5 ± 13.84	46.36 ± 13.44	57.06 ± 15.12	0.854	0.980
**LDL**	129.86 ± 31.06	116.68 ± 31.17	129.53 ± 27.91	105.72 ± 29.95	127.73 ± 39.17	117.94 ± 45.54	0.932	0.143
**Cholesterol**	204.8 ± 33.81	196.13 ± 41.21	210.07 ± 31.19	179.64 ± 38.93	210.47 ± 50.08	194.06 ± 54.34	0.865	0.142
**Triglycerides**	166.05 ± 54.84	110.19 ± 50.7	188.26 ± 101.59	101.85 ± 52.47	198.66 ± 106.71	108.92 ± 49.4	0.381	0.634
**Insulin**	38.13 ± 41.62	9.5 ± 5.45	37.93 ± 39.66	10.57 ± 6.39	44.37 ± 47.31	11.88 ± 8.24	0.599	0.290

The data are presented as mean ± standard deviation. *p*: paired samples *T*-test and one-way analysis of variance (ANOVA)

**Table 6:** Comparison of postoperative complications and re-admissions based on type of surgery.

		Type of Surgery			*p*
		RNYGB (n = 20)	MGB (n = 62)	SG (n = 129)	
**Re-admissions**	**Ileus**	1	0	2	0.1
	**Blood replacement**	2	1	1	
	**Nausea and vomiting**	1	1	7	
	**Incisional hernia**	0	3	0	
**Postoperative Complications**	**Atelectasis**	1	1	1	0.781
	**Leakage**	0	1	4	
	**Pneumonia**	0	0	2	
	**Surgical site infections**	0	2	0	
	**Hemorrhage**	0	1	2	

The data are presented as n. Test: Chi-square test.

## Data Availability

All data generated or analyzed during this study are included in this published article.
